# Insights into food preference in hybrid F1 of *Siniperca chuatsi* (♀) × *Siniperca scherzeri* (♂) mandarin fish through transcriptome analysis

**DOI:** 10.1186/1471-2164-14-601

**Published:** 2013-09-05

**Authors:** Shan He, Xu-Fang Liang, Jian Sun, Ling Li, Ying Yu, Wei Huang, Chun-Mei Qu, Liang Cao, Xiao-Li Bai, Ya-Xiong Tao

**Affiliations:** 1 Key Laboratory of Freshwater Animal Breeding, Ministry of Agriculture, College of Fisheries, Huazhong Agricultural University, Hubei Collaborative Innovation Center for Freshwater Aquaculture, Wuhan 430070, PR China; 2 Department of Anatomy, Physiology, and Pharmacology, College of Veterinary Medicine, Auburn University, Auburn, AL 36849-5519, USA

**Keywords:** Food preference, Live prey fish, Mandarin fish, Transcriptome sequencing, Digital gene expression

## Abstract

**Background:**

As economically relevant traits, feeding behavior and food preference domestication determine production cost and profitability. Although there are intensive research efforts on feeding behavior and food intake, little is known about food preference. Mandarin fish accept only live prey fish and refuse dead prey fish or artificial diets. Very little is currently known about the genes regulating this unique food preference.

**Results:**

Using transcriptome sequencing and digital gene expression profiling, we identified 1,986 and 4,526 differentially expressed genes in feeders and nonfeeders of dead prey fish, respectively. Up-regulation of Crbp, Rgr and Rdh8, and down-regulation of Gc expression, consistent with greater visual ability in feeders, could promote positive phototaxis. Altered expressions of period, casein kinase and Rev-erbα might reset circadian phase. Down-regulation of orexigenic and up-regulation of anorexigenic genes in feeders were associated with lower appetite. The mRNA levels of Creb, c-fos, C/EBP, zif268, Bdnf and Syt were dramatically decreased in feeders, which might result in significant deficiency in memory retention of its natural food preference (live prey fish). There were roughly 100 times more potential SNPs in feeders than in nonfeeders.

**Conclusions:**

In summary, differential expression in the genes identified shed new light on why mandarin fish only feed on live prey fish, with pathways regulating retinal photosensitivity, circadian rhythm, appetite control, learning and memory involved. We also found dramatic difference in SNP abundance in feeders vs nonfeeders. These differences together might account for the different food preferences. Elucidating the genes regulating the unique food preference (live prey fish) in mandarin fish could lead to a better understanding of mechanisms controlling food preference in animals, including mammals.

## Background

As economically relevant traits, feeding behavior and food preference domestication determine production cost and profitability. Although there are intensive research efforts on feeding behavior and food intake [1,2], little is known about food preference. Food preference is an innate behavioral trait subject to genetic influences. However, the genes and genetic factors that determine food preference are largely unknown. Although several genes, including genes encoding taste receptors such as Tas1r1 [3,4], PKD2L1, PKD1L3 and CD36 [5], obesity-associated genes such as FTO [6] and APOA2 [7], and metabolism genes such as AMY1 [8] and RNASE1 [9], were shown to be involved in the determination of food preference, very little is currently known about the transcriptome determining the unique food preference, such as live prey.

Mandarin fish, as a demersal piscivore, is found only in the freshwaters of China and the River Amur along the Russian borderlands. It has very unique food preference. In the wild, once the fry start feeding, it feed solely on live fry of other fish species [10]. In rearing conditions, mandarin fish also accept only live prey fish, refusing dead prey fish or artificial diets [11]. Among major species of mandarin fish, *Siniperca scherzeri*, is much easier to accept dead prey fish compared to *S. chuatsi*, and the difference in acceptance of dead prey fish is further amplified in the hybrid F1 of *S. chuatsi* (♀) × *S. scherzeri *(♂). We hypothesize that changes in gene expression as well as SNPs account for this dramatic difference. Elucidating the genes regulating the unique food preference (live prey fish) in mandarin fish could lead to a better understanding of mechanisms controlling food preference in animals, including mammals.

In higher vertebrates such as mammals, the responses of primary centers are coordinated by correlation centres, and the cerebrum is the site of memory and considers the results of experiences on which intelligence and learning are based. In lower vertebrates such as fish, sense organs directly send signals to the primary centers to initiate behavior. Hence their behavior consists of reflex responses with little variation or modification by experience. Therefore compared with mammals, the study of feeding behavior and food preference in fish could enable us to observe direct effects of genes and genetic factors on food preference. To elucidate the relationship between gene expression and food preference, we performed de novo transcriptome sequencing and digital gene expression profiling (DGE) of feeders and nonfeeders of dead prey fish in mandarin fish. We showed that expression of genes in several pathways, including retinal photosensitivity, circadian rhythm, appetite control, learning and memory, were significantly different in feeders and nonfeeders. These potential determinants provide a glimpse of genetic architecture of the unique food preference.

## Results

### High-throughput sequencing and annotation of unigenes

To obtain an overview of gene expression profile in mandarin fish with different food preferences, cDNA libraries were constructed from dead prey fish feeders (SC_X) and nonfeeders (SC_W), and sequenced using the Illumina Hiseq2000 system. High quality reads of SC_X and SC_W were assembled, yielding 665,466, and 716,044 contigs, respectively (Table 1). After removing the partial overlapping sequences, a total of 118,218 distinct sequences were obtained (All-Unigene, mean size: 506 bp, N50: 611 bp) (Additional file 1). Among these unigenes, 69.5% (82,108) were between 100 and 500 bp in length, 30.5% (36,110) were longer than 500 bp, of which 9.8% (11,550) were longer than 1,000 bp. Six unigenes were aligned with either Sanger-derived sequences that we obtained by sequencing PCR products or reference sequences deposited in NCBI. Each sequence had more than 98% coverage validation (Additional files 2, 3, 4, 5, 6, 7). The sequencing data in this study have been deposited in the EBI ArrayExpress database (accession number: E-MTAB-1365).

**Table 1:** Summary of data generated from mandarin fish transcriptome

	**SC_X**			**SC_W**		
	**Reads (n)**	**Bases (Mb)**	**Average length(bp)**	**Reads (n)**	**Bases (Mb)**	**Average length(bp)**
Clean reads	25,558,980	2300	-	22,681,824	2041	-
Contigs	665,466	94.7	142	716,044	101.1	141
Scaffold	173,329	53.7	310	181,076	56.7	313
Unigene	122,998	47.6	387	127,174	50.1	394
	Number		Bases (Mb)		Average length(bp)	
All-Unigene	118,218		59.8		506	

49,155 unigenes (41.6% of All-Unigene) were aligned function by BLASTx, and 69,063 (58.4%) assembled sequences could not be matched to any known protein. 48,796 annotated sequences had significant matches with 27,354 unique accession numbers. The BLASTx top-hit species distribution of gene annotations showed highest homology to zebrafish (*Danio rerio*, 52%), followed by Atlantic salmon (*Salmo salar*, 9%) and spotted green pufferfish (*Tetraodon nigroviridis*, 4%) (Table 2). In addition, the mandarin fish sequences also had homologies to four other fish species including Japanese pufferfish (*Takifugu rubripes*), sablefish (*Anoplopoma fimbria*), rainbow smelt (*Osmerus mordax*) and medaka (*Oryzias latipes*). These results indicated a high level of phylogenetic conservation between mandarin fish and other fish species, especially zebrafish. 182 unigenes of our transcriptome libraries matched the published mandarin fish protein sequences (645) currently available in NCBI database, suggesting the identification of a large number of new genes by transcriptome sequencing reported here.

**Table 2:** **Species distribution of unigene BLASTX results with a cutoff E value < 10**^**-5 **^**and the proportions of each species**

**Species name**	**Number of**	**Percent of**
	**blast hits**	**blast hits**
*Danio rerio*	25350	52%
*Salmo salar*	4470	9.1%
*Tetraodon nigroviridis*	1712	3.4%
*Xenopus (Silurana) tropicalis*	1472	3%
*Gallus gallus*	1110	2.3%
*Takifugu rubripes*	1102	2.2%
*Anoplopoma fimbria*	909	1.8%
*Osmerus mordax*	641	1.3%
*Mus musculus*	592	1.2%
*Oryzias latipes*	483	0.98%

**Figure 1:**
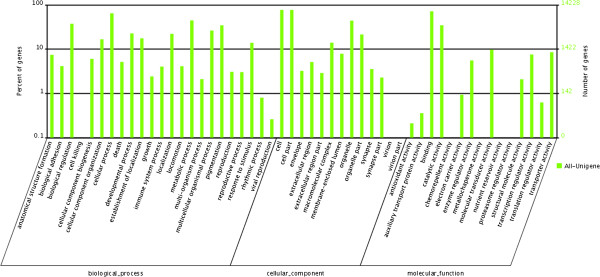
**Functional annotation of mandarin fish transcripts based on GO categorization.** The left y-axis indicates the percentage of a specific category of genes in that main category. The right y-axis indicates the number of genes in a category.

Of 49,155 annotated sequences in mandarin fish transcriptome, 14,228 (28.9%) were assigned with one or more gene ontology (GO) terms. In total, 106,024 GO assignments were finally obtained, with 46.1% for biological processes, 34.8% for cellular components, and 19.1% for molecular functions. Level 2 GO functional categories were summarized in Figure 1. The number and assortment of allocated GO categories reflected the diversity and complexity of genes expressed in mandarin fish. We mapped the 49,155 annotated sequences to the reference canonical pathways in Kyoto Encyclopedia of Genes and Genomes (KEGG) to identify the biological pathways. In total, we assigned 30,964 sequences to 205 known metabolic or signaling pathways. The representative pathways with the differentially expressed genes were mitogen-activated protein kinases (MAPK) signaling pathway (1222 members), calcium signaling pathway (913 members), insulin signaling pathway (711 members), long-term potentiation (LTP) (542 members), long-term depression (369 members), taste transduction (173 members) and mammalian circadian rhythm (92 members) (Additional file 8).

### SNP and SSR discovery

The transcript/EST-based markers are important resource for determining functional genetic variation [12]. We detected 4,768 potential SNPs in feeders and 41 potential SNPs in nonfeeders. The overall frequency of predicted SNPs in the mandarin fish transcriptome was one per 12,430 bp. A total of 4,809 SNPs were identified, including 1,592 transitions and 3,217 transversions; 2,510 of these SNPs had been annotated. Of these SNPs, 2,062 were identified from unigenes covered by ten or more reads, suggesting that 42.9% of SNPs found in this transcriptome were covered with sufficient sequencing depth and more likely to represent ‘true’ SNPs. Of 19 SNP loci predicted to reside in the 10 amplified sequences, 15 sites were validated (Additional file 9). There were roughly 100 times more potential SNPs in feeders than in nonfeeders. Whether the difference in SNP prevalence accounts for the different acceptance of dead prey fish in hybrid F1 warrants further investigation. For identification of Simple Sequence Repeats (SSRs), all 118,218 unigenes generated in this study were applied to determine potential microsatellites using Batchprimer3.0 (http://probes.pw.usda.gov/batchprimer3/index.html). We identified a total of 22,418 potential SSRs in 17,933 (15.2%) unigenes with frequency of one SSR per 2.7 kb of the unigenes. Of 17,933 SSR-containing sequences, 7,585 (42.3%) had been annotated, and could be considered as preferred candidates for marker development.

### Identification of differentially expressed genes

**Figure 2:**
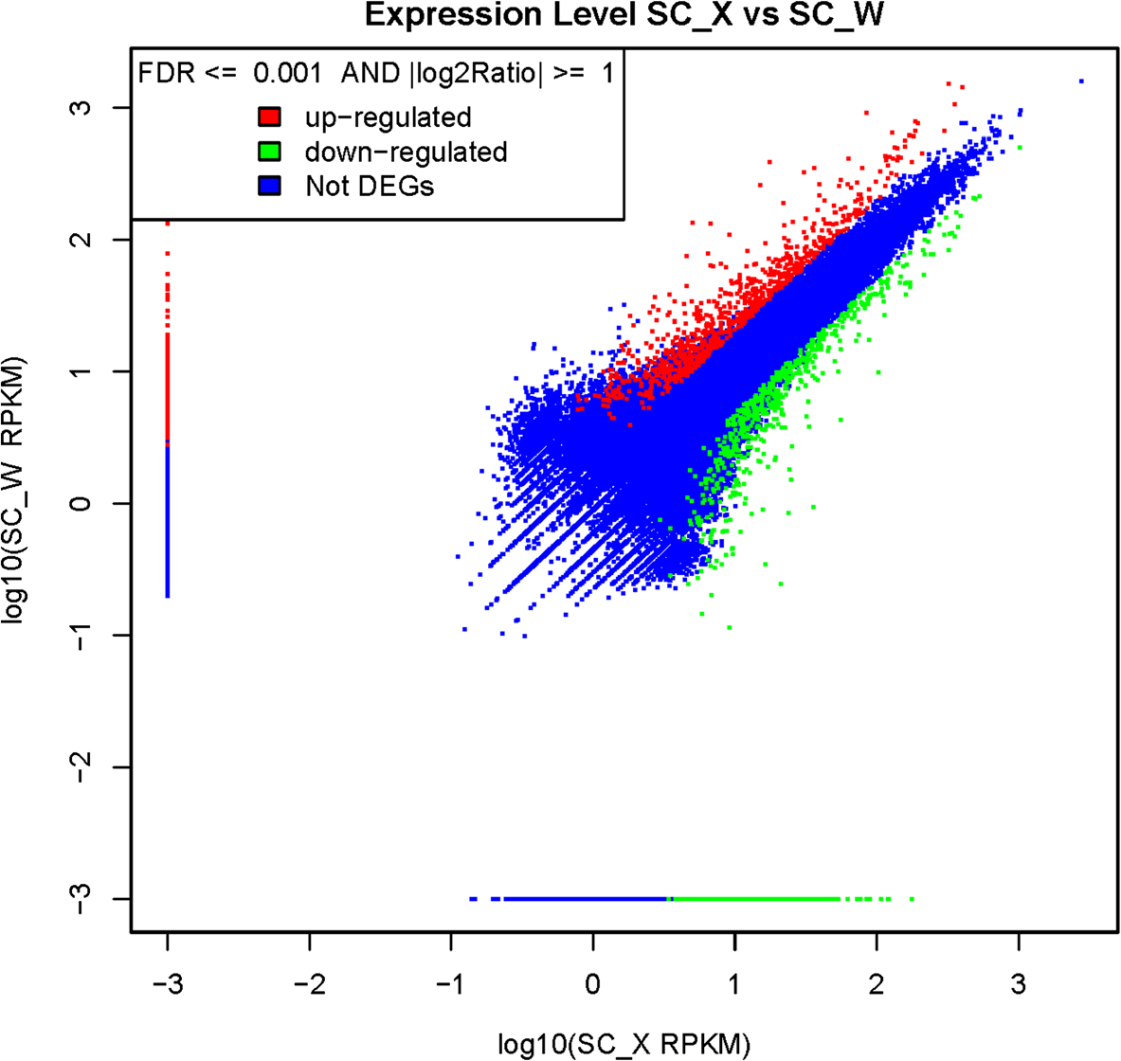
**Scatter plot showing the correlation between the expression levels of feeders and nonfeeders by transcriptome sequencing.** SC_X and SC_W indicate feeders and nonfeeders, respectively. The x-axis contains Log10 of Reads Per Kb per Million reads (RPKM) of feeders and the y-axis indicates Log10 of RPKM of nonfeeders. Limitations were based on FDR ≤ 0.001, and the absolute value of Log2 (SC_X / SC_W) ≥ 1.

**Figure 3:**
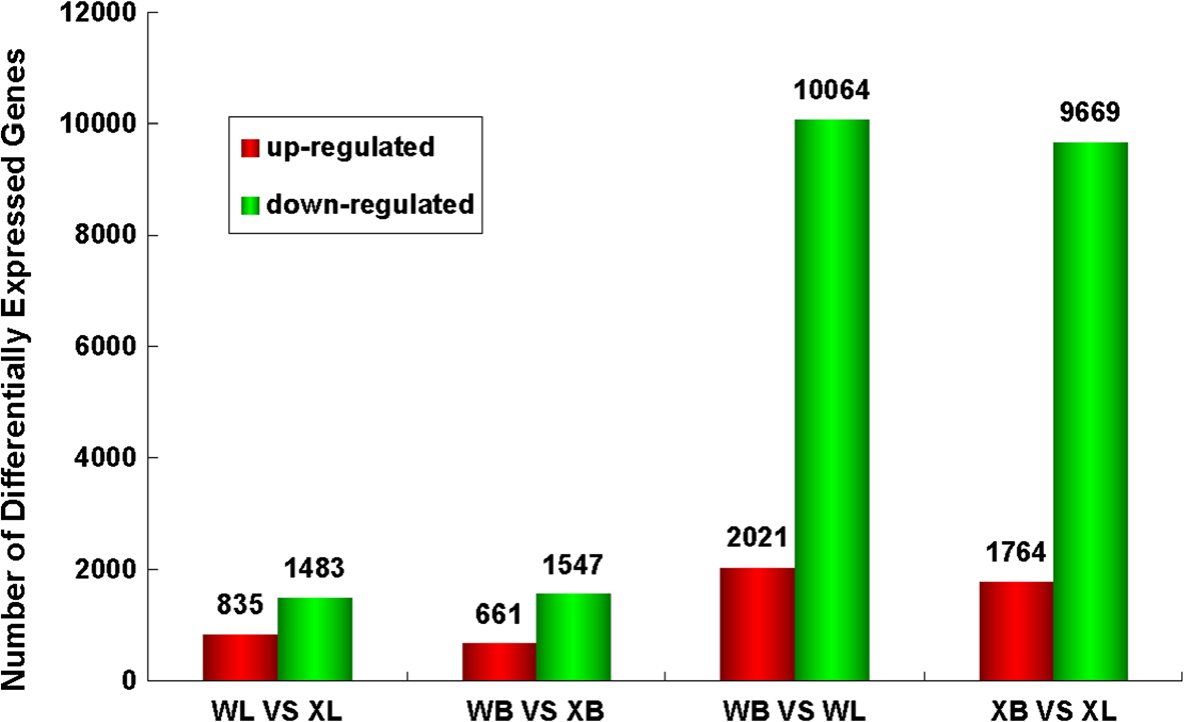
**Differential expression analysis of unigenes by DGE.** WL and WB indicate liver and brain in nonfeeders, respectively, while XL and XB indicate liver and brain in feeders. The superscripts of each column represent the number of differentially expressed genes between groups.

We found 1,986 and 4,526 unigenes to be differentially expressed between feeders and nonfeeders from transcriptome and DGE analysis (False Discovery Rate (FDR) ≤ 0.001, fold-change ≥ 2, Figures 2 and 3), respectively. Analysis of these genes revealed the signaling pathways involved, including retinal photosensitivity (retinal G protein-coupled receptor (Rgr), retinol dehydrogenase 8 (Rdh8), cellular retinol-binding protein (Crbp) and guanylate cyclase (Gc)), circadian rhythm (period 1 (Per1), period 2 (Per2), Rev-erbα, casein kinase (Ck) and nocturnin), appetite control (neuropeptide Y (Npy), growth hormone (Gh), pro-opiomelanocortin (Pomc), peptide YY (Pyy), insulin and leptin), learning and memory (cyclic AMP-response element-binding protein (Creb), c-fos, fos-related antigen 2 (Fra-2), CCAAT enhancer binding protein (C/EBP), zif268, brain-derived neurotrophic factor (Bdnf) and synaptotagmin (Syt)) (Additional file 10, Figure 4). The importance of these genes was further supported by the identification of significant potential SNP, SSR and antisense transcripts in these genes. To compare the two tissues (liver and brain) that are of interest for food preference, we identified 11,433 unigenes that were differentially expressed between liver and brain in feeders, and 12,085 unigenes in nonfeeders (Figure 3) by DGE. In addition, DGE analysis of mandarin fish with the two different food preferences generated 9,597,700 clean tags from brain and 9,964,672 clean tags from liver, respectively (Additional file 11).

**Figure 4:**
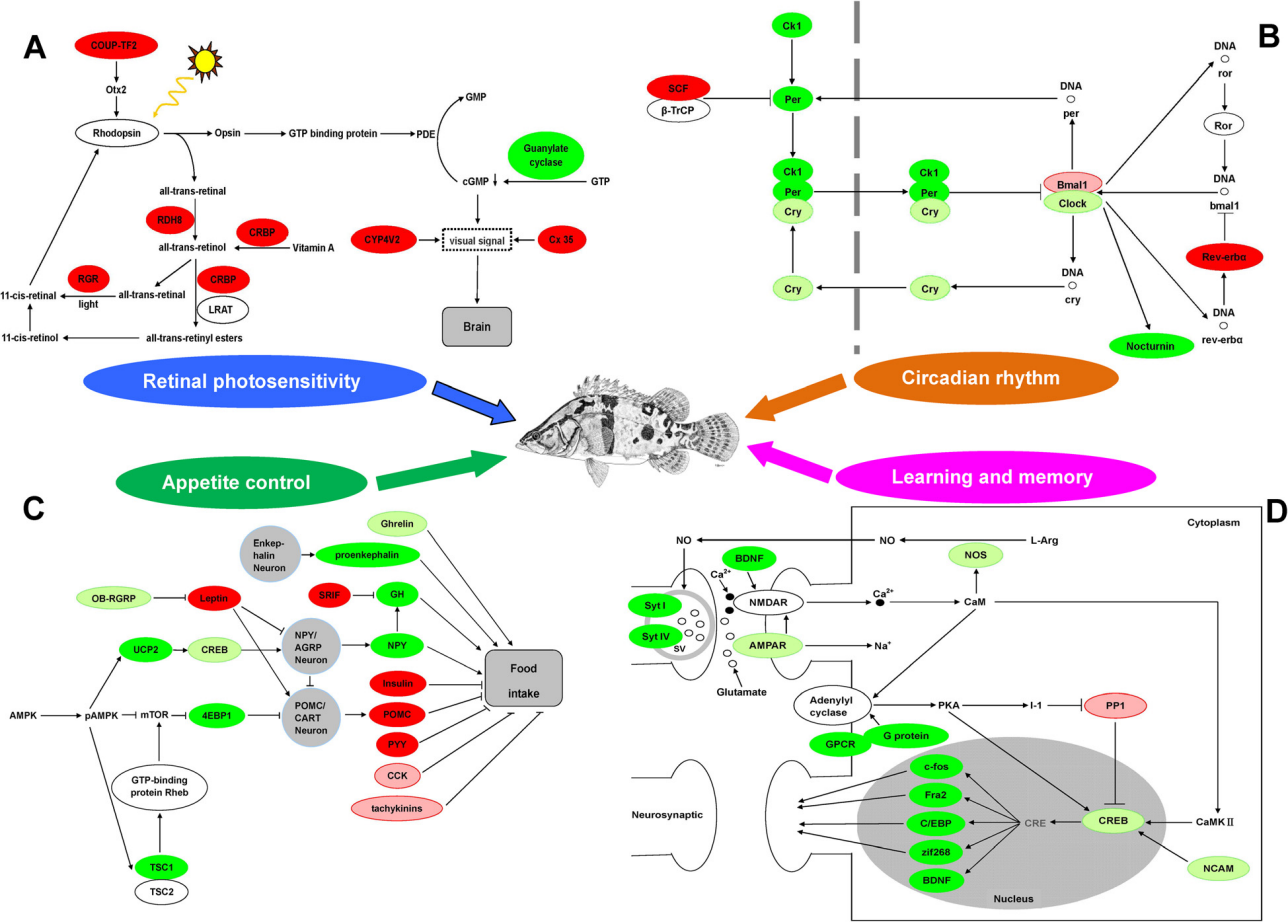
**Differentially expressed genes between feeders and nonfeeders from transcriptome and DGE analysis.** The most important pathways related to live prey food preference included retinal photosensitivity **(A)**, circadian rhythm **(B)**, appetite control **(C)**, learning and memory **(D)**. The colors of ellipses were shaded according to significance level (bright red: the mRNA expression levels of feeders were significantly higher than those in nonfeeders (FDR ≤ 0.001, the absolute value of log2[Ratio] ≥ 1); pink: the mRNA expression levels of feeders were slightly higher than those in nonfeeders (FDR ≤ 0.5, the absolute value of log2[Ratio] ≥ 0.5); bright green: the mRNA expression levels of feeders were significantly lower than those in nonfeeders (FDR ≤ 0.001, the absolute value of log2[Ratio] ≥ 1); pale green: the mRNA expression levels of feeders were slightly lower than those in nonfeeders (FDR ≤ 0.5, the absolute value of log2[Ratio] ≥ 0.5).

**Figure 5:**
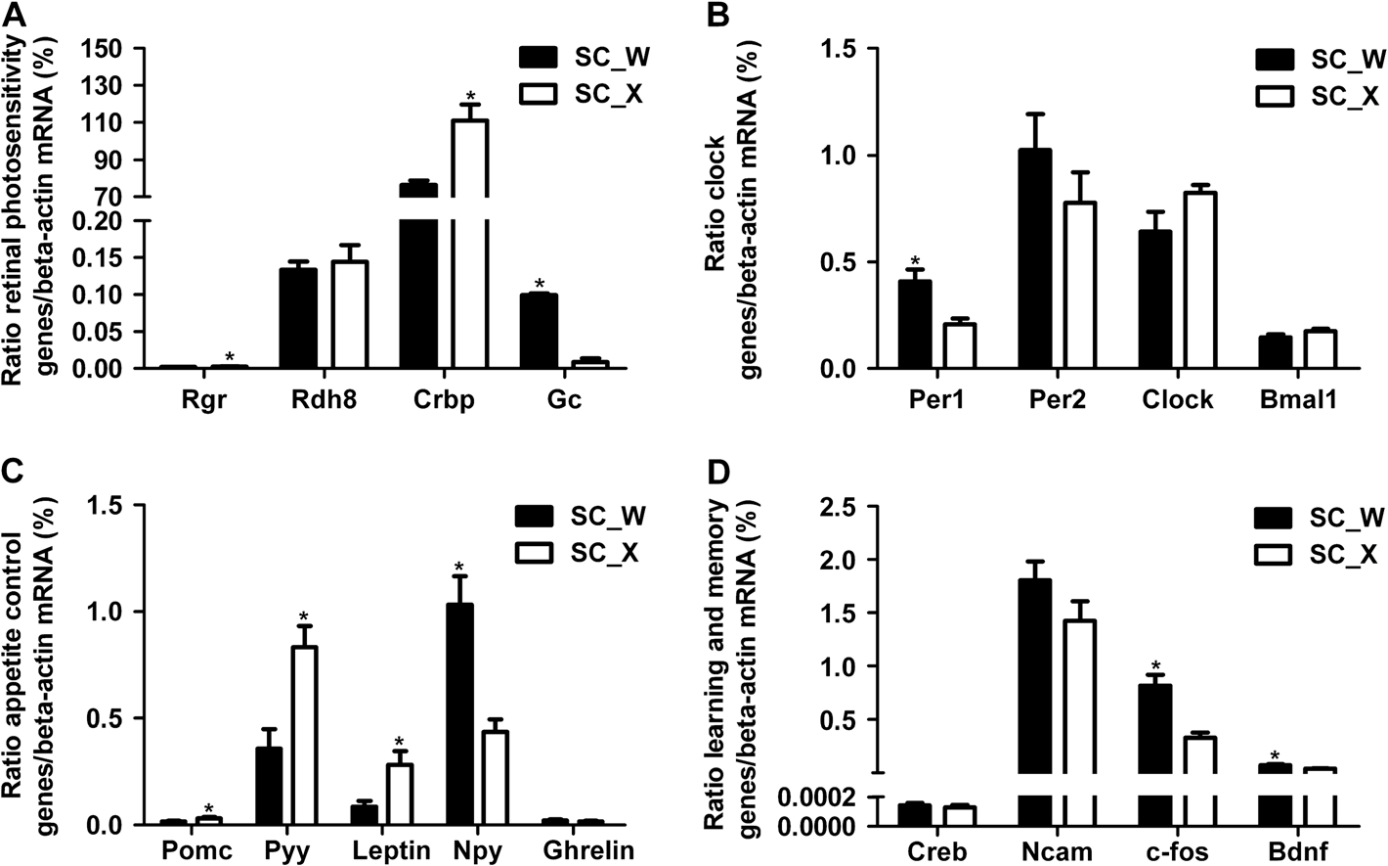
**Validation of differentially expressed genes with Real-time RT-PCR. A**. The relative mRNA abundance of retinal photosensitivity genes (Rgr, Rdh8, Crbp and Gc) in feeders and nonfeeders was determined by Real-time RT-PCR. Compared with nonfeeders, the mRNA levels of Rgr and Crbp were significantly increased, whereas Gc was decreased significantly. **B**. The relative mRNA abundance of clock genes (Per1, Per2, Clock and Bmal1) in feeders and nonfeeders was determined by Real-time RT-PCR. Compared with nonfeeders, the mRNA levels of Per1 and Per2 were decreased significantly and slightly, respectively, whereas Clock and Bmal1 were slightly increased. **C**. The relative mRNA abundance of appetite control genes (Pomc, Pyy, leptin, Npy and ghrelin) in feeders and nonfeeders was determined by Real-time RT-PCR. Compared with nonfeeders, the mRNA levels of Pomc, Pyy and leptin were significantly increased, whereas Npy and ghrelin were decreased significantly and slightly, respectively. **D**. The relative mRNA abundance of learning and memory genes (Creb, Ncam, c-fos and Bdnf) in feeders and nonfeeders was determined by Real-time RT-PCR. Compared with nonfeeders, the mRNA levels of Creb and Ncam were slightly decreased, c-fos and Bdnf were decreased significantly. Data are presented as mean ± standard error (n = 6). * indicates significant differences between groups based on one-way analysis of variance (ANOVA) followed by the post hoc test (P < 0.05).

Real-time RT-PCR was frequently used to confirm data obtained from high-throughput sequencing [13,14]. Here, we also used Real-time RT-PCR to confirm the differential expression of 18 genes in *S. chuatsi* that went through the same training procedure. The data obtained were consistent with those obtained from the transcriptome sequencing and DGE analysis (Figure 5).

## Discussion

Little is known about the genes and biological mechanisms controlling food preference in animals. In this study, by profiling the transcriptomes of dead prey fish feeders and nonfeeders in mandarin fish, we identified differentially expressed genes potentially influencing the unique food preference of live prey, including those affecting retinal photosensitivity, circadian rhythm, appetite control, learning and memory. Real-time RT-PCR confirmed the differential expression in selected genes. We also found dramatic difference in SNP abundance in feeders vs nonfeeders. These differences together might account for the different food preferences.

### Differentially expressed genes involved in retinal photosensitivity pathway

Animals make food choices based on a number of physiological, nutritional, environmental, and sociocultural factors [5]. Sensitivity of sensory system is critical to food preference. It is thus advantageous for mandarin fish to catch prey fish at night through the perception of motion and shape with the help of its well-developed scotopic vision [11]. Although *Salmo spp.* also shows the selection of food motion and shape to some extent, the offered food pellet can be captured immediately before it falls down to the bottom of the tank because they have high visual acuity, thus can feed swiftly by darting [15-17]. This contrasts with mandarin fish, which has low visual acuity and feed only by stalking. Mandarin fish might not be able to accomplish its relatively long process of prey recognition before the offered food pellet has fallen down to the bottom of the tank and can no longer be perceived by its sensory organs. The more strict selection of prey motion and shape also makes it more difficult to feed mandarin fish with artificial diets and dead prey fish. Hence much better developed visual ability could improve the mandarin fish to accept dead prey fish or artificial diets [11].

Adaptation to dark in most animals is associated with increased 11-cis-retinal generation and rhodopsin reconstitution. Factors that interfere with the rhodopsin cycle or its downstream signaling pathways will affect vision, especially scotopic vision . We observed differential expression of Crbp, Rgr, Rdh8 and Gc in brains of feeders vs nonfeeders. Crbp is involved in the initial processing of retinol from food [18]. Light-dependent formation of 11-cis-retinal by the retinal pigment epithelium and regeneration of rhodopsin under photic conditions involve the RGR opsin located in the retinal pigment epithelium [19]. RDH8 is a critical regulator of chromophore regeneration [20]. Gc, as a family of enzymes that catalyze the conversion of GTP to cGMP, are central in phototransduction cascade [21]. Our results suggested that the up-regulation of Crbp, Rgr and Rdh8, and down-regulation of Gc in feeders might lead to increased 11-cis-retinal and rhodopsin levels, deceased cGMP generation, leading to greater visual ability and light sensitivity. Thus the feeders could capture the dead prey fish before it falls to the bottom of the tank. Moreover, the mRNA expression levels of connexin 35 (Cx35), Cyp4v2 and chicken ovalbumin upstream promoter transcription factor 2 (Coup-tf2) genes in the brain of feeders were all higher than those of nonfeeders, potentially contributing to the food preference [22-25] (Figure 4A).

### Differentially expressed genes involved in circadian rhythm pathway

Previous studies demonstrated that the molecular mechanisms of circadian rhythm generation in zebrafish appear to be generally consistent with the mammalian model [26]. We identified homologs of the mammalian clock genes in mandarin fish. We found differential expression in several clock genes, including Per1, Per2, cryptochrome (Cry), Clock, Bmal1, CkIα, CkIγ, CkIδ, CkIIβ, Rev-erbα, Skp1 and Rbx2 in feeders vs nonfeeders (Figure 4B). These genes are known to be critical regulators of circadian rhythm [27-33]. Taken together, changes in expression levels of these clock genes in feeders might reset circadian phase and contribute to the unique food preference.

### Differentially expressed genes involved in appetite control pathway

Previous studies provide a framework for understanding the regulation of food intake in mammals and fish. Peripheral signals such as leptin from adipocytes, insulin from endocrine pancreas, cholecystokinin and peptide YY from gastrointestinal tract are incorporated in the hypothalamus to generate orexigenic (such as NPY and ghrelin) or anorexigenic (such as α-melanocyte stimulating hormone (α-MSH) derived from POMC) signals [34]. We observed lower expression of orexigenic genes (Npy, proenkephalin, Gh, uncoupling protein 2 (Ucp2), Creb, eukaryotic initiation factor (eIF) 4E binding protein (4Ebp1), tuberous sclerosis 1 (Tsc1), ghrelin and leptin receptor gene-related protein (Ob-rgrp)) and higher expression of anorexigenic genes (Pomc, Pyy, preprosomatostatin (Srif), insulin, leptin, cholecystokinin (Cck) and tachykinin 1) in feeders compared with nonfeeders (Figure 4C). These genes are well established regulators of energy homeostasis and play important roles in determination of food preferences [35-46]. The changes in gene expression suggested that feeders had decreased appetite.

### Differentially expressed genes involved in learning and memory pathway

Food habits can develop with learning experience [47,48]. Our previous study indicated that sensory modality and associative learning appear to be critical factors in food discrimination of mandarin fish [49]. However, study on genes involved in learning and memory of nocturnal piscivorous fish has received little attention. A number of molecules participate in learning and memory processes. The TORC1-mediated CREB regulation is a critical molecular step underlying synaptic plasticity and long-term memory [50,51]. As a suppressor of CREB, protein phosphatase 1 (PP1) determines the efficacy of learning and memory by limiting acquisition and favoring memory decline [52]. Neural cell adhesion molecule (NCAM) plays an important role in axonal growth, learning, and memory through activating the phosphorylation of MAPKs and CREB [53]. In our study, compared with nonfeeders, the mRNA levels of Creb and Ncam were dramatically decreased and Pp1 were slightly increased in feeders, protentially resulting in significant deficiency in the maintenance of long-term memory of its natural food preference, and therefore feeders were able to accept novel food (dead prey fish) (Figure 4D).

Along the same line, as members of immediate early gene and the Fos family of transcription factors, c-fos and Fra-2 mRNA expressions are up-regulated in response to a variety of neuronal activation protocols, including LTP [54-56]. In the current study, c-fos, Fra-2 and zif268 genes were expressed at significantly lower levels in feeders than in nonfeeders, suggesting disruption of LTP and memory formation in feeders [57]. We hypothesize that the acquisition of novel food preference might be closely associated with effacement of the original memory of natural food preference (live prey), and the enhanced learning capacity for new food preference (dead prey). We also showed that in mandarin fish, a number of genes necessary for memory formation and retention (C/EBP, Bdnf, Syt I, Syt IV and nitric oxide synthase (Nos)) were differentially expressed in feeders vs nonfeeders. They might also be involved in the determination of the unique food preference in mandarin fish (Figure 4D) [58-61].

## Conclusions

In summary, our results showed that there were individual differernces in hybrid F1 of mandarin fish in their feeding response to dead prey fish. The acquisition of novel food preference (dead prey fish) might be due to enhanced visual ability, resetting of circadian phase, decreased appetite, deficiency in memory retention and more abundant variant alleles. Interaction of retinal photosensitivity, circadian rhythm, appetite control, learning and memory outputs might drive the feeding behavior.

## Methods

### Fish and sample preparation

The hybrid F1 of *S. chuatsi* (♀) × *S. scherzeri* (♂) (body length about 5 cm) were obtained from Guangdong Freshwater Fish Farm (Panyu, Guangdong Province, China). The training was performed for 3 days following the methods previously described by Liang et al. [49] using net-cages as the experimental culture. Fry of India mrigal *Cirrhina mrigola* were used as the live prey fish in this study, and the dead prey fish were frozen India mrigal fry. During the training period, the fish were visually sorted into feeders and nonfeeders on the basis of plumpness or emaciation, respectively. The training period did not cause the nonfeeders to starve to death because of the relatively short time and large size of the fish. To eliminate the influence of hunger on the mRNA levels of mandarin fish, both groups were fed with live prey fish for two days after training. And then 16 fish were randomly selected from each group. Total RNA was isolated from brain and liver tissues using SV total RNA isolation system (Promega, Madison, WI, USA) according to manufacturer's protocol. Equal amount of total RNA from brain and liver tissues of 16 fish in each group were pooled and used to construct the libraries for transcriptome and DGE analysis. The animal protocol was approved by Huazhong Agricutural University.

### Transcriptome library preparation and Illumina sequencing

The samples for transcriptome analysis were prepared using Illumina's kit following manufacturer's instructions (San Diego, CA, USA). Poly(A) mRNA was purified from total RNA (a mixture of equal amount of RNA from brain and liver) using oligo-dT-attached magnetic beads. Paired-end cDNA libraries were sequenced using Illumina HiSeq2000 system. Image deconvolution and base calling were performed with the Illumina CASAVA v1.7. The reliability of the reads was 89.1% with average length of the reads at 90 bp. The library construction and sequencing were performed by the commercial service provider Beijing Genomics Institute at Shenzhen (Shenzhen, China). Transcriptome assembly was carried out with short reads assembling program SOAPdenovo [62] with k-mer length from 21 to 41 bp. Then the reads are mapped back to assembled contigs. By using the paired-end information, it is able to detect contigs from the same transcript as well as the distances between these contigs. We connected the contigs using N to represent unknown sequences between each pair of contigs, and then scaffolds were made. Paired-end reads were used again for gap filling of scaffolds to obtain sequences with least Ns and could not be extended on either end. Such sequences were defined as unigenes. Separate cDNA libraries were constructed from dead prey fish feeders (SC_X) and nonfeeders (SC_W), unigenes from each library were taken into further process of sequence splicing and redundancy removing with sequence clustering software to acquire non-redundant unigenes (All-Unigene) as long as possible. The assembly parameters were more than 89.1% identity over a minimum of 50 bases with a maximum of 2 bases of unmatched overhangs at sequence end.

To annotate the mandarin fish transcriptome, we performed the BLASTx alignment (e-value < 0.00001) between All-Unigene and protein databases such as NCBI, Swiss-Prot, KEGG and COG. Functional annotation by gene ontology terms (GO; http://www.geneontology.org) was accomplished with Blast2GO software. After obtaining GO annotation for each unigene, we applied WEGO software [63] to conduct GO functional classification for all unigenes. To assess the quality of sequencing data, 3 unigenes generated by transcriptome sequencing were randomly selected for cloning and sequence validation by PCR. All three PCR products were sequenced using an ABI Prism^TM^ 377 (PerkinElmer, Waltham, MA, USA). To ascertain the quality of SOAPdenovo assembly of short reads into long contiguous RNA transcript sequences, we aligned 3 of the transcriptome-derived unigenes to the corresponding Sanger-sequenced, full-length, cloned mRNA sequences from mandarin fish in NCBI.

### SSR and SNP analysis

To detect SNPs in the cDNA pool, the consensus assembly sequence generated from the two trancriptome libraries was employed as a reference sequence to which individual reads were aligned using SOAPsnp [64]. SNP identification was limited to the unigenes containing at least five reads. The minimum allele quality (accumulated sequence quality for every allele) was not lower than 20. We considered only SNPs, excluding all indels and variants involving more than one nucleotide. To validate the detected SNPs, we designed primers to amplify 10 unigenes containing potential SNPs. The PCR products were sequenced with both forward and reverse primers using ABI Prism^TM^ 377 (PerkinElmer). Batchprimer3.0 was adopted to identify and localize microsatellite motifs, which were defined as di-nucleotide SSR with a minimum of six repetitions, four repetitions for trinucleotide, and three repetitions for tetra- to hexa-nucleotide motifs.

### Digital gene expression profiling

The four tag libraries of mandarin fish (the liver tissues of feeders and nonfeeders, the brain tissues of feeders and nonfeeders, respectively) were constructed in parallel using the Digital Gene Expression Tag Profile Kit (Illumina) according to the manufacturer's instructions. Six μg of total RNA per sample was used for mRNA capture using oligo-dT magnetic bead adsorption and oligo-dT was used to synthesize the first and second-strand cDNA. The 5' ends of tags were generated by endonuclease NlaIII, which recognizes and cuts off the CATG sites on cDNA. The cDNA fragments with 3' ends were then purified with magnetic beads and Illumina adapter 1 was ligated to the sticky 5' ends. The junction of Illumina adapter 1 and CATG site is the recognition site of MmeI, which cuts 17 bp downstream of the CATG site, producing tags with adapter 1. After removing 3' fragments with magnetic beads precipitation, the 21-bp unique tags with adaptor 1 were purified and ligated to adaptor 2 to form a cDNA tag library. Sequencing by synthesis (SBS) was performed using the Illumina HiSeq2000 system. All high quality tags were mapped to the reference sequence generated by transcriptome sequencing, and only 1 bp mismatch was permitted. For gene expression analysis, the number of unambiguous clean tags for each gene was calculated and then normalized to TPM (number of transcripts per million tags) [65].

### Identification of differentially expressed genes

Gene expression levels were measured through short reads mapping in Reads Per Kb per Million reads (RPKM) [66]. GO functional analysis and KEGG pathway analysis were then carried out in differentially expressed genes. SYBR Green Real-time RT-PCR was performed to validate the transcriptome and DGE data (Additional file 12). In addition to the hybrid F1 of *S. chuatsi* (♀) × *S. scherzeri* (♂), total RNA was prepared from *S. chuatsi* that went through the same training procedure and used for the validation of differentially expressed genes. Beta-actin was amplified in parallel as an internal control. There were six biological and three technical replicates respectively.

### Statistical analysis

We used FDR ≤ 0.001 and the absolute value of log2[Ratio] ≥ 1 as the threshold to judge the significance of gene expression difference. Statistical analysis was performed with SPSS13.0 software. Data normality and homogeneity of variances were analyzed. Results were presented as the means ± S.E. (n = 6) for each group. One-way analysis of variance (ANOVA) followed by the post hoc test were carried out to determine whether the differences between groups were significant (P < 0.05).

## Competing interests

The authors declare that they have no competing interests.

## Authors’ contributions

SH, JS and LL performed the de novo assembly and bioinformatical analysis. SH, YY and LC contributed to the fish and sample preparation. WH, CMQ and XLB contributed to the RT-PCR and SNP experiments. XFL gave technical advice and contributed to the study design. SH, XFL and YXT co-wrote the paper. All authors read and approved the final manuscript.

## Supplementary Material

Additional file 1 **Length distribution of All-Unigene.** (> 200 bp, mean length = 506 bp, N50 = 611 bp, Max = 8,514 bp). Click here for file

Additional file 2 **Alignment of de novo assembled unigene (Unigene5395_All) with a Sanger-derived sequence.** Multiple alignments were performed by ClustalW and DNAMAN. The hatched region shows the matched sequence, and coverage validation is 99.13%. Click here for file

Additional file 3 **Alignment of de novo assembled unigene (Unigene13401_All) with a Sanger-derived sequence.** Multiple alignments were performed by ClustalW and DNAMAN. The hatched region shows the matched sequence, and coverage validation is 100%. Click here for file

Additional file 4 **Alignment of de novo assembled unigene (Unigene26407_All) with a Sanger-derived sequence.** Multiple alignments were performed by ClustalW and DNAMAN. The hatched region shows the matched sequence, and coverage validation is 98.76%. Click here for file

Additional file 5 **Alignment of de novo assembled unigene (Unigene41802_All) with reference sequence deposited in NCBI.** Insulin-like growth factor-1 (Genbank HM164110.1). Multiple alignments were performed by ClustalW and DNAMAN. The hatched region shows the matched sequence, and coverage validation is 99.56%. Click here for file

Additional file 6 **Alignment of de novo assembled unigene (Unigene56717_All) with reference sequence deposited in NCBI.** Insulin-like growth factor-2 (Genbank HM164111.1). Multiple alignments were performed by ClustalW and DNAMAN. The hatched region shows the matched sequence, and coverage validation is 98.04%. Click here for file

Additional file 7 **Alignment of de novo assembled unigene (Unigene58882_All) with reference sequence deposited in NCBI.** Tropomyosin (Genbank JN165713.1). Multiple alignments were performed by ClustalW and DNAMAN. The hatched region shows the matched sequence, and coverage validation is 99.74%. Click here for file

Additional file 8 Representative pathways involved in food preference determination in mandarin fish. Click here for file

Additional file 9 Validation of potential SNPs detected in mandarin fish transcriptome. Click here for file

Additional file 10 The differentially expressed genes involved in food preference determination of mandarin fish. Click here for file

Additional file 11 A comparison of the number of digital tags generated from the brain and liver libraries. Click here for file

Additional file 12 Primer sequences for Real-time RT-PCR. Click here for file
