# Supplementary figures and images for: Insights into food preference in hybrid F1 of Siniperca chuatsi (♀) × Siniperca scherzeri (♂) mandarin fish through transcriptome analysis
Source: BMC Genomics. 2013 Sep 5;14:601. doi: 10.1186/1471-2164-14-601 (PMC3846499; doi:10.1186/1471-2164-14-601)

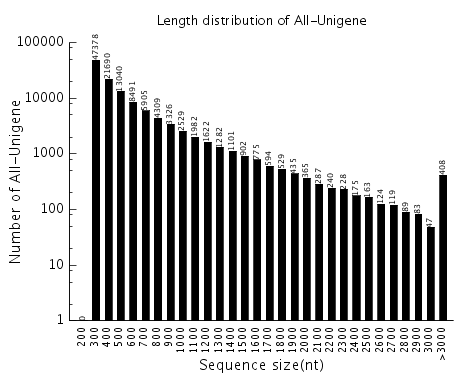

Supplement: Additional file 1 — Length distribution of All-Unigene. (> 200 bp, mean length = 506 bp, N50 = 611 bp, Max = 8,514 bp). [file 1471-2164-14-601-S1.tiff]

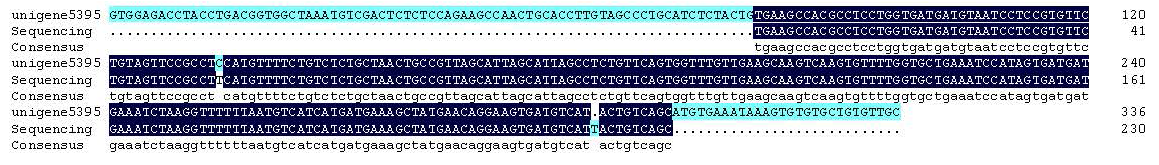

Supplement: Additional file 2 — Alignment of de novo assembled unigene (Unigene5395_All) with a Sanger-derived sequence. Multiple alignments were performed by ClustalW and DNAMAN. The hatched region shows the matched sequence, and coverage validation is 99.13%. [file 1471-2164-14-601-S2.tiff]

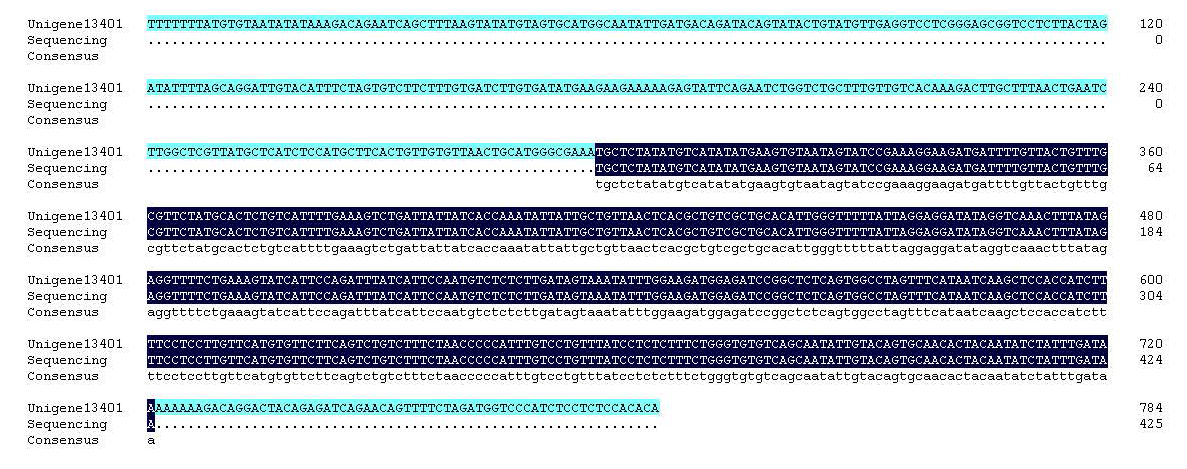

Supplement: Additional file 3 — Alignment of de novo assembled unigene (Unigene13401_All) with a Sanger-derived sequence. Multiple alignments were performed by ClustalW and DNAMAN. The hatched region shows the matched sequence, and coverage validation is 100%. [file 1471-2164-14-601-S3.tiff]

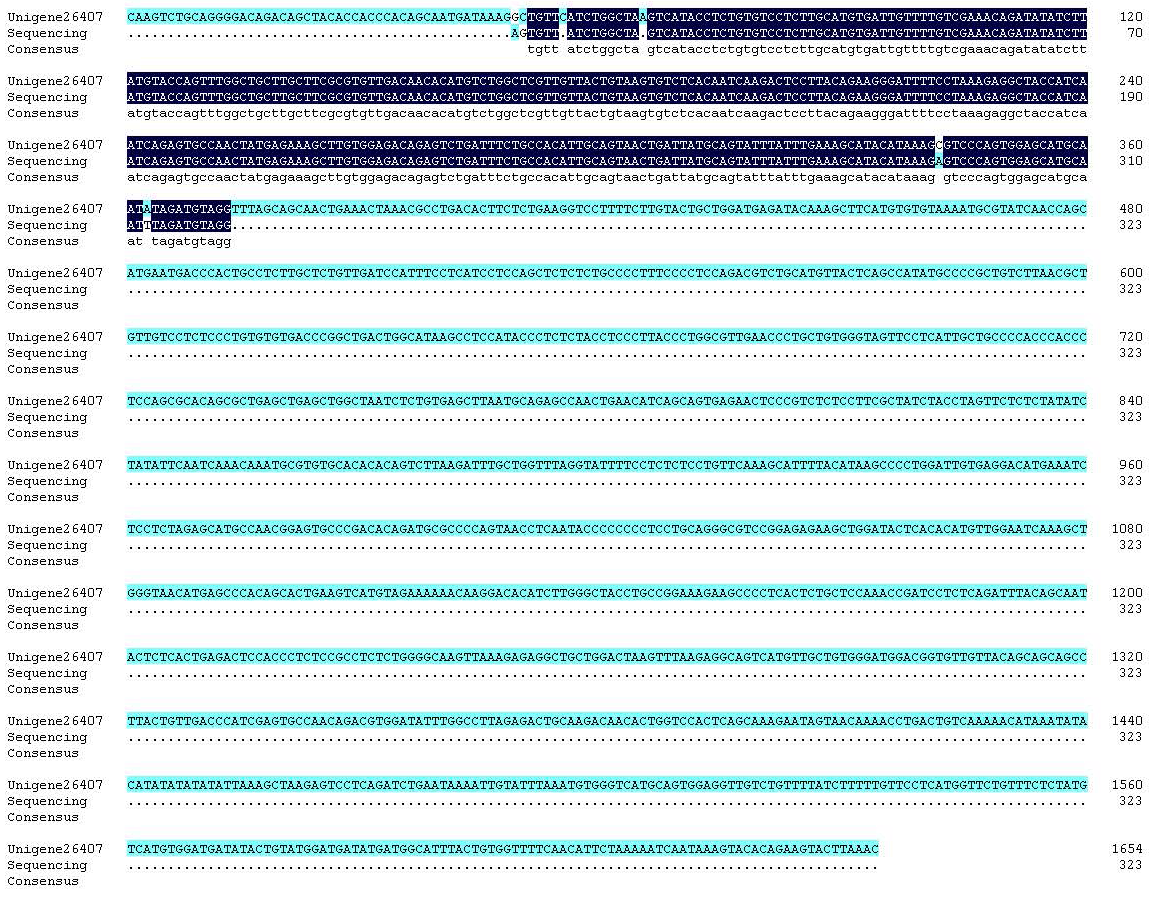

Supplement: Additional file 4 — Alignment of de novo assembled unigene (Unigene26407_All) with a Sanger-derived sequence. Multiple alignments were performed by ClustalW and DNAMAN. The hatched region shows the matched sequence, and coverage validation is 98.76%. [file 1471-2164-14-601-S4.tiff]

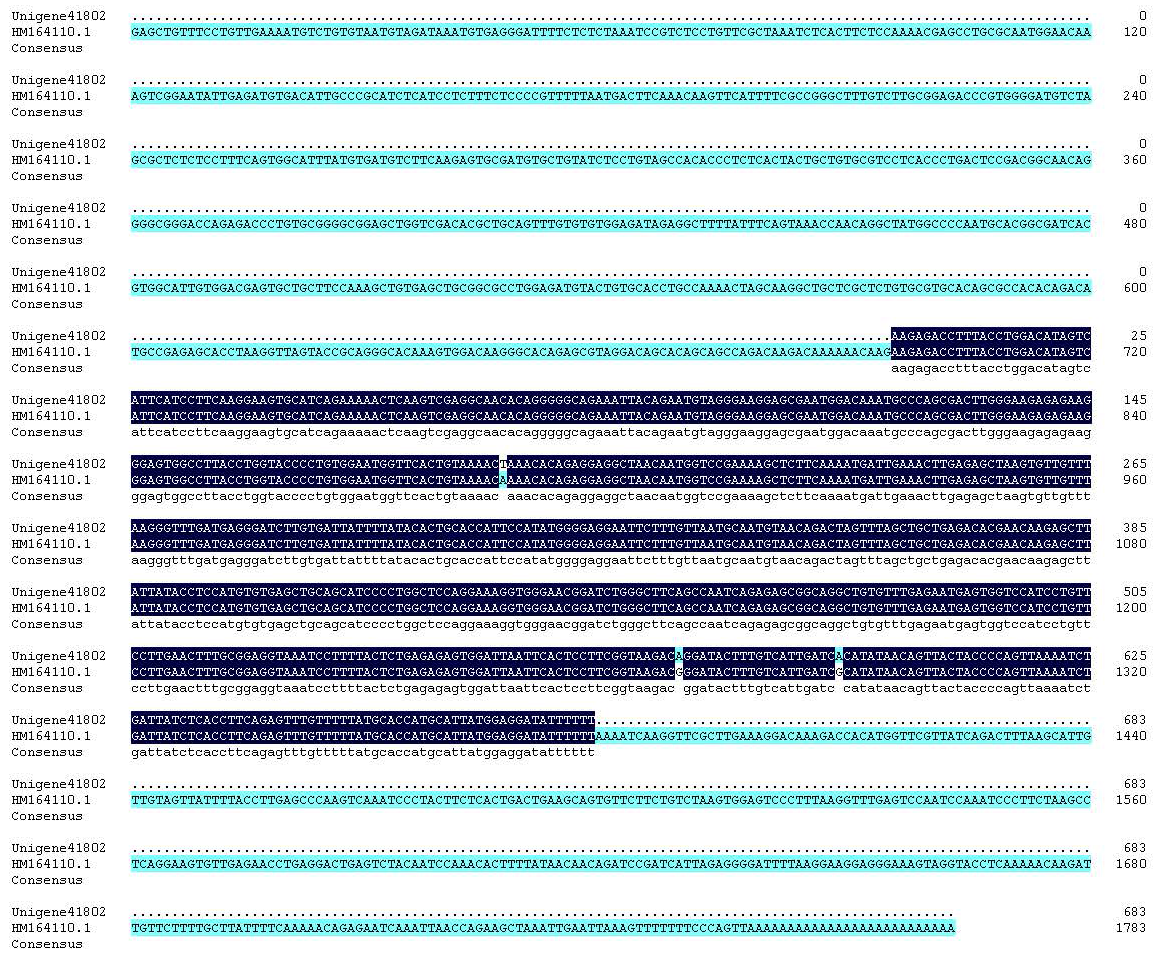

Supplement: Additional file 5 — Alignment of de novo assembled unigene (Unigene41802_All) with reference sequence deposited in NCBI. Insulin-like growth factor-1 (Genbank HM164110.1). Multiple alignments were performed by ClustalW and DNAMAN. The hatched region shows the matched sequence, and coverage validation is 99.56%. [file 1471-2164-14-601-S5.tiff]

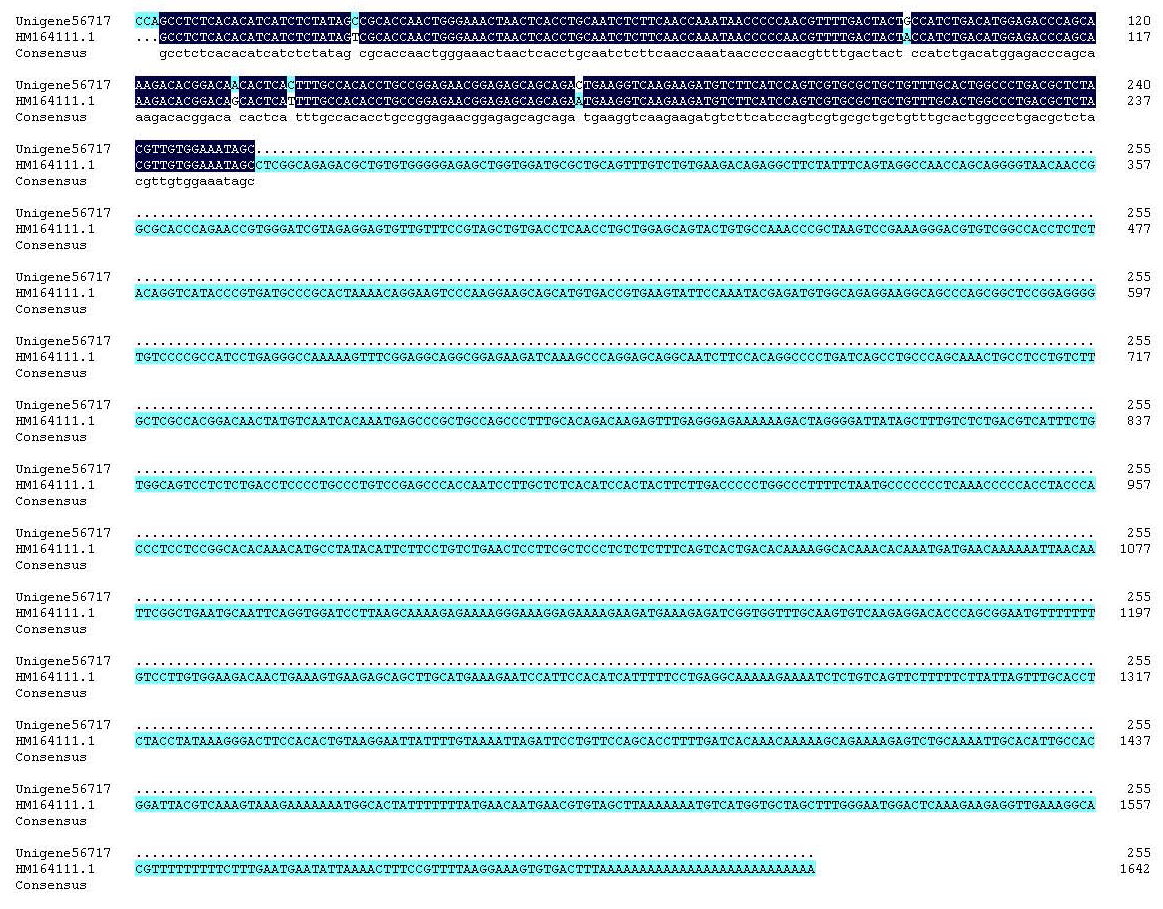

Supplement: Additional file 6 — Alignment of de novo assembled unigene (Unigene56717_All) with reference sequence deposited in NCBI. Insulin-like growth factor-2 (Genbank HM164111.1). Multiple alignments were performed by ClustalW and DNAMAN. The hatched region shows the matched sequence, and coverage validation is 98.04%. [file 1471-2164-14-601-S6.tiff]

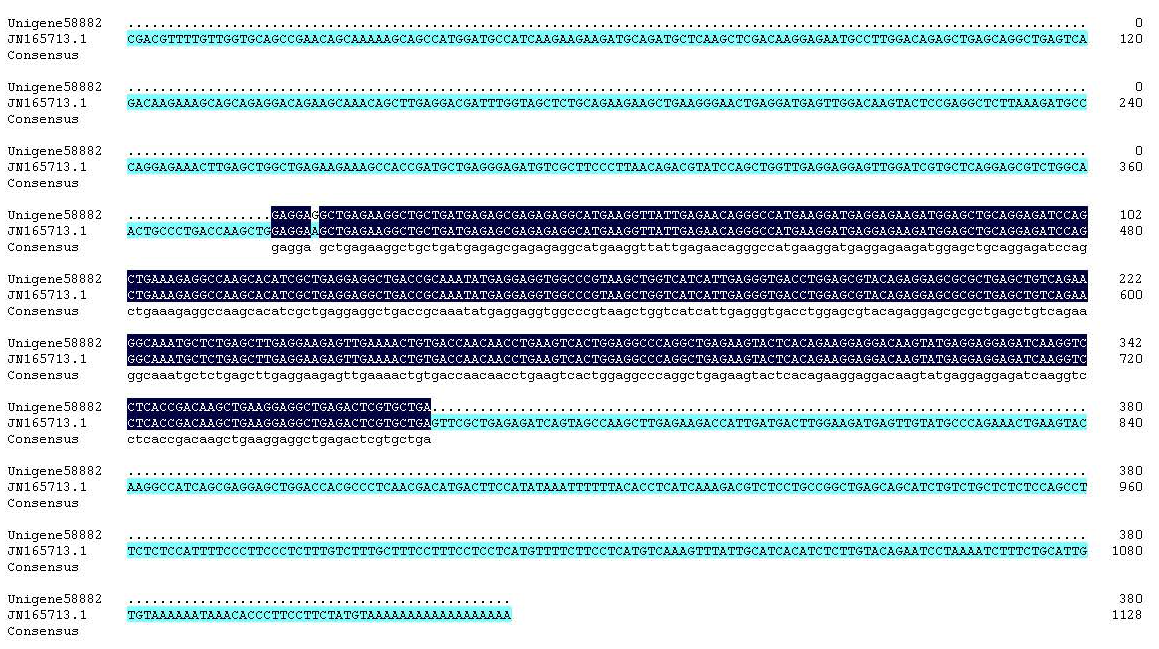

Supplement: Additional file 7 — Alignment of de novo assembled unigene (Unigene58882_All) with reference sequence deposited in NCBI. Tropomyosin (Genbank JN165713.1). Multiple alignments were performed by ClustalW and DNAMAN. The hatched region shows the matched sequence, and coverage validation is 99.74%. [file 1471-2164-14-601-S7.tiff]
