# Supplementary material for: Insights into food preference in hybrid F1 of Siniperca chuatsi (♀) × Siniperca scherzeri (♂) mandarin fish through transcriptome analysis
Source: BMC Genomics. 2013 Sep 5;14:601. doi: 10.1186/1471-2164-14-601 (PMC3846499; doi:10.1186/1471-2164-14-601)
Supplement: Additional file 8 — Representative pathways involved in food preference determination in mandarin fish. [file 1471-2164-14-601-S8.doc]

Additional file 8. Representative pathways involved in food preference determination in mandarin fish.

| Pathway | All-Unigene | | differentially expressed genes from transcriptome | | differentially expressed genes from DGE | |
| --- | --- | --- | --- | --- | --- | --- |
|  | Number of members | percentage | Number of members | percentage | WL VS XL | WB VS XB |
| MAPK signaling pathway | 1222 | 4.0% | 21 | 3.0% | 22 | 24 |
| Calcium signaling pathway | 913 | 3.0% | 21 | 3.0% | 12 | 7 |
| Insulin signaling pathway | 711 | 2.3% | 25 | 3.6% | 22 | 8 |
| Long-term potentiation | 542 | 1.8% | 5 | 0.7% | 9 | 6 |
| GnRH signaling pathway | 505 | 1.6% | 10 | 1.4% | 9 | 11 |
| ErbB signaling pathway | 502 | 1.6% | 8 | 1.2% | 8 | 9 |
| Melanogenesis | 474 | 1.5% | 8 | 1.2% | 8 | 8 |
| Long-term depression | 369 | 1.2% | 6 | 0.9% | 6 | 7 |
| Olfactory transduction | 317 | 1.0% | 7 | 1.0% | 4 | 2 |
| Apoptosis | 280 | 0.9% | 10 | 1.4% | 8 | 4 |
| Taste transduction | 173 | 0.6% | 1 | 0.1% | 1 | 1 |
| Circadian rhythm-mammal | 92 | 0.3% | 10 | 1.4% | 0 | 1 |
| Circadian rhythm-fly | 71 | 0.2% | 9 | 1.3% | 1 | 1 |
