# Supplementary material for: Insights into food preference in hybrid F1 of Siniperca chuatsi (♀) × Siniperca scherzeri (♂) mandarin fish through transcriptome analysis
Source: BMC Genomics. 2013 Sep 5;14:601. doi: 10.1186/1471-2164-14-601 (PMC3846499; doi:10.1186/1471-2164-14-601)
Supplement: Additional file 9 — Validation of potential SNPs detected in mandarin fish transcriptome. [file 1471-2164-14-601-S9.doc]

Additional file 9. Validation of potential SNPs detected in mandarin fish transcriptome.

| Gene ID | | Number of potential SNPs | | Number of true SNPs | Annealing temperature（℃） | Sequence of primers (5′–3′) | | PCR product length (bp) |
| --- | --- | --- | --- | --- | --- | --- | --- | --- |
| Unigene13401_All  Unigene13707_All  Unigene26407_All    Unigene26663_All    Unigene31920_All  Unigene39621_All  Unigene40751_All  Unigene46170_All  Unigene51384_All  Unigene63424_All | | 4  1  1  1  3    1  1  1  1  5 | | 4  1  0  1  1  1  1  1  1  4 | 56  60  56  56  60  60  55  60  60  56 | Forward: GGCTCGTTATGCTCATCTCCA  Reverse: ACTGTTCTGATCTCTGTAGTCCT  Forward: ACCAACAGCCAGCCAATG  Reverse: CAAACCGCTGCCACGAT  Forward: GGACAGACAGCTACACCACC  Reverse: GAGCATGCAATATAGATGTAGG  Forward: CACTATGCTGCTGCTTCTGCCTT  Reverse: CACTGGGCACTGAGGGATAATG  Forward: GTGTTGATGAAACCCTGATGT  Reverse: CTCTTCCTCCCTCTGTCCTT  Forward: TATCGTCTCCGTCGTCCTCT  Reverse: GAGCCACAATGTTGACGAGG  Forward: GCAGTGCACTCCAAGGAAAAGA  Reverse: AACACAGGGTTGCCTTCTGC  Forward: TAGAAGATGGAGAAGGTGGTG  Reverse: TGCTGTTTCCAAGGAGGTA  Forward: ATGTGTGTAGCTGTTAGTCG  Reverse: CAGTTATGAGGCACTATACAG  Forward: TGCCATCTTGACCACCAGAGAAC  Reverse: ATGGCGTCCATCTGACAGATTCG | | 523  497  361  775  156  237  341  138  270  515 |
| Total | 19 | | 15 | |  |  |  | |
