# Supplementary material for: Insights into food preference in hybrid F1 of Siniperca chuatsi (♀) × Siniperca scherzeri (♂) mandarin fish through transcriptome analysis
Source: BMC Genomics. 2013 Sep 5;14:601. doi: 10.1186/1471-2164-14-601 (PMC3846499; doi:10.1186/1471-2164-14-601)
Supplement: Additional file 10 — The differentially expressed genes involved in food preference determination of mandarin fish. [file 1471-2164-14-601-S10.doc]

Additional file 10. The differentially expressed genes involved in food preference determination of mandarin fish.

| Unigene ID | | Gene name | SSR | SNP | Antisense Transcripts |
| --- | --- | --- | --- | --- | --- |
| Retinal photosensitivity | | | | | |
| 30 | retinal G protein-coupled receptor (Rgr) | |  |  | Y |
| 3294 | retinol dehydrogenase 8 (Rdh8) | |  |  | Y |
| 87956 | cellular retinol-binding protein (Crbp) | |  |  |  |
| 13282 | guanylyl cyclase (Gc) | |  |  | Y |
| 51601 | guanylyl cyclase (Gc) | |  |  | Y |
| 43549 | Chicken ovalbumin upstream promoter transcription factor 2 (Coup-tf2) | | Y |  |  |
| 76153 | connexin 35 (Cx35) | |  |  |  |
| 53812 | Cyp4v2 | |  |  |  |
| Circadian rhythm | | | | | |
| 37992 | period 1 (Per1) | |  |  | Y |
| 51244 | period 1 (Per1) | |  |  | Y |
| 52370 | period 2 (Per2) | | Y |  |  |
| 118154 | period 2 (Per2) | | Y |  |  |
| 117709 | period 2 (Per2) | |  |  |  |
| 117718 | period 2 (Per2) | |  |  |  |
| 54335 | cryptochrome-1-like (Cry) | |  |  | Y |
| 581 | Clock | | Y |  |  |
| 26034 | Bmal1 | |  |  |  |
| 650 | Rev-erbα | |  |  |  |
| 59021 | Rev-erbα | |  |  | Y |
| 14640 | casein kinase I isoform delta-like (CkIα) | | Y |  |  |
| 28925 | casein kinase I isoform gamma-like (CkIγ) | |  |  |  |
| 59637 | casein kinase I isoform alpha-like (CkIδ) | |  |  | Y |
| 818 | Skp1 | |  |  | Y |
| 9217 | Rbx2 | |  |  | Y |
| 41936 | nocturnin | |  |  | Y |
| Appetite control | | | | | |
| 86634 | neuropeptide Y (Npy) | |  |  |  |
| 3077 | proenkephalin | |  |  | Y |
| 5395 | growth hormone (Gh) | |  |  | Y |
| 101453 | uncoupling protein 2 (Ucp2) | | Y |  | Y |
| 52462 | cyclic AMP-response element-binding protein (Creb) | | Y |  |  |
| 38439 | cyclic AMP-response element-binding protein (Creb) | |  |  | Y |
| 52013 | eukaryotic initiation factor (eIF) 4E binding protein (4Ebp1) | |  |  | Y |
| 117596 | tuberous sclerosis 1 (Tsc1) | |  |  |  |
| 94305 | ghrelin | | Y |  |  |
| 38410 | leptin receptor gene-related protein (Ob-rgrp) | | Y |  | Y |
| 103248 | pro-opiomelanocortin (Pomc) | |  |  | Y |
| 57335 | pro-opiomelanocortin (Pomc) | |  |  | Y |
| 34952 | pro-opiomelanocortin (Pomc) | |  |  | Y |
| 3731 | peptide YY (Pyy) | | Y |  | Y |
| 33330 | preprosomatostatin II (Srif) | |  |  | Y |
| 50012 | preprosomatostatin I (Srif) | | Y |  | Y |
| 60326 | preprosomatostatin III (Srif) | |  |  | Y |
| 45774 | insulin | | Y |  | Y |
| 110017 | leptin | |  |  |  |
| 39550 | cholecystokinin (Cck) | |  |  | Y |
| 51384 | tachykinin 1 | |  | Y | Y |
| Learning and memory | | | | | |
| 52462 | cyclic AMP-response element-binding protein (Creb) | | Y |  |  |
| 38439 | cyclic AMP-response element-binding protein (Creb) | |  |  | Y |
| 25636 | c-fos | |  |  | Y |
| 7588 | fos-related antigen 2 (Fra-2) | | Y |  | Y |
| 39632 | CCAAT enhancer binding protein (C/EBP) | | Y |  | Y |
| 42292 | CCAAT enhancer binding protein (C/EBP) | |  |  | Y |
| 56663 | CCAAT enhancer binding protein (C/EBP) | |  |  |  |
| 58666 | CCAAT enhancer-binding protein (C/EBP) | |  |  | Y |
| 59876 | zif268 | | Y |  | Y |
| 775 | brain-derived neurotrophic factor (Bdnf) | |  |  | Y |
| 38825 | neural cell adhesion molecule (Ncam) | | Y | Y |  |
| 16120 | serine/threonine-protein phosphatase PP1 | | Y | Y | Y |
| 12976 | nitric oxide synthase (Nos) | |  |  | Y |
| 35344 | synaptotagmin I (Syt I) | |  |  | Y |
| 29207 | synaptotagmin IV (Syt IV) | | Y | Y | Y |
| 5161 | probable glutamate receptor-like | |  |  | Y |
| 31 | G-protein coupled receptor family C group 5 member B | | Y |  | Y |
| 27026 | G protein-coupled receptor 3 | |  |  | Y |
| 49422 | G protein-coupled receptor 137 | |  |  |  |
| 53654 | G protein subunit gamma 5 | |  |  | Y |

Y indicates the existence of potential SNP, SSR or antisense transcripts in the genes.
