# Supplementary material for: Insights into food preference in hybrid F1 of Siniperca chuatsi (♀) × Siniperca scherzeri (♂) mandarin fish through transcriptome analysis
Source: BMC Genomics. 2013 Sep 5;14:601. doi: 10.1186/1471-2164-14-601 (PMC3846499; doi:10.1186/1471-2164-14-601)
Supplement: Additional file 11 — A comparison of the number of digital tags generated from the brain and liver libraries. [file 1471-2164-14-601-S11.doc]

Additional file 11. A comparison of the number of digital tags generated from the brain and liver libraries.

| Libraries | Clean Tag | All Tag Mapped to Gene | | All Tag-mapped Genes | |
| --- | --- | --- | --- | --- | --- |
|  | Total number | Total number | Distinct Tag number | number | percentage |
| WL | 4862124 | 1716674 | 43692 | 21288 | 18.0% |
| XL | 5102548 | 1799108 | 42279 | 21571 | 18.3% |
| WB | 4875740 | 2611908 | 84573 | 35500 | 30.0% |
| XB | 4721960 | 2501544 | 75334 | 33810 | 28.6% |
