# Supplementary material for: Insights into food preference in hybrid F1 of Siniperca chuatsi (♀) × Siniperca scherzeri (♂) mandarin fish through transcriptome analysis
Source: BMC Genomics. 2013 Sep 5;14:601. doi: 10.1186/1471-2164-14-601 (PMC3846499; doi:10.1186/1471-2164-14-601)
Supplement: Additional file 12 — Primer sequences for Real-time RT-PCR. [file 1471-2164-14-601-S12.doc]

Additional file 12. Primer sequences for Real-time RT-PCR.

|  | Gene name | Sequence of the primers (5′-3′) | Accession number |
| --- | --- | --- | --- |
| Internal control gene | Beta-actin | Forward: AGAGGGAAATCGTGCGTGAC | GenBank: AY885683.1 |
|  |  | Reverse: ATACCGAGGAAGGAAGGCTG |  |
| Retinal photosensitivity genes | Rgr | Forward: CTGGAGCACTACTCTGACGAT | Unigene30_All |
|  |  | Reverse: TGGTGAAAGCTGGGAACAT |  |
|  | Rdh8 | Forward: AGCAGTGTCATGGGTCTTC | Unigene3294_All |
|  |  | Reverse: ATGTCCTCTGGCATCTGG |  |
|  | Crbp | Forward: CGACATTGACTTTGCCACC | Unigene87956_All |
|  |  | Reverse: CACTGTTTCCAGCCACGAT |  |
|  | Gc | Forward: CACATCACTCCAATGCAGGT | Unigene13282_All |
|  |  | Reverse: GGCATCTCCAATAGTCTCAACC |  |
| Clock genes | Per1 | Forward: ACTGTTCTTTCCAACCCTGTG | Unigene51244_All |
|  |  | Reverse: TGTGGCTTGCTGTGATGC |  |
|  | Per2 | Forward: CGCATCTTCACCACCACA | Unigene117718_All |
|  |  | Reverse: GCCCGCATACTTCAACACT |  |
|  | Clock | Forward: GCTGGAACAGAGGACAAGA | Unigene581_All |
|  |  | Reverse: TTTGCGGGACATGATTG |  |
|  | Bmal1 | Forward: CAAGATTGGACGCATGATTG | Unigene26034_All |
|  |  | Reverse: GGACTGGTTGGAGTAGGGATAG |  |
| Appetite control genes | Pomc | Forward: TGTTAGTGGTGGTGATGGC | Unigene34952_All |
|  |  | Reverse: CCTGTCGCTGTGGGCTTT |  |
|  | Pyy | Forward: GGAGTCGTCGTATCTTGATC | Unigene3731_All |
|  |  | Reverse: CGCCCTGAGACACTACATTA |  |
|  | Leptin | Forward: TGAAGTGGATGGCTGAAC | Unigene110017_All |
|  |  | Reverse: CTACCTTGACCTGGGAGAC |  |
|  | Npy | Forward: CAGCCCTGAGACACTACAT | Unigene86634_All |
|  |  | Reverse: AGAGGCAGGGTGGTCATT |  |
|  | Ghrelin | Forward: TTTGCTGGTCTTCCTGTTGTG | Unigene75761_All |
|  |  | Reverse: ATGTGGTTGTCCTCAGTGGGT |  |
| Learning and memory genes | Creb | Forward: TTCTCCCTAATCCTGTTCCC | Unigene52462_All |
|  |  | Reverse: TTCCCATCAGTGCGGTAAT |  |
|  | Ncam | Forward: GCTGCTGGGATACAAGATCTAC | Unigene38825_All |
|  |  | Reverse: CCTCGCTCTAATCTCTCCTCTT |  |
|  | c-fos | Forward: GGGGCAGAATGGAACAGAT | Unigene25636_All |
|  |  | Reverse: AGGGATGGGCAGGAGAAA |  |
|  | Bdnf | Forward: CTGATAAGAGCCAGGGAGG | Unigene775_All |
|  |  | Reverse: CCAGAAGAAAGAGCAACGG |  |
